# Estrogen and progesterone receptor testing in breast carcinoma: concordance of results between local and reference laboratories in Brazil

**DOI:** 10.1590/S1516-31802011000400007

**Published:** 2011-05-05

**Authors:** Sheila Cristina Lordelo Wludarski, Lisandro Ferreira Lopes, Ívison Xavier Duarte, Filomena Marino Carvalho, Lawrence Weiss, Carlos Eduardo Bacchi

**Affiliations:** I MD. Associate Pathologist, Pathology Consultancy, Botucatu, São Paulo, Brazil.; II MD, PhD. Associate Professor, Department of Pathology, Faculdade de Medicina da Universidade de São Paulo (FMUSP), São Paulo, Brazil.; III MD, PhD. Chair, Division of Pathology, City of Hope National Medical Center, Duarte, California, United States.; IV MD, PhD. Director and Chief Pathologist, Pathology Consultancy, Botucatu, São Paulo, Brazil.

**Keywords:** Breast neoplasms, Receptors, estrogen, Receptors, progesterone, Immunohistochemistry, Brazil, Neoplasias da mama, Receptores estrogênicos, Receptores de progesterona, Imunoistoquímica, Brasil

## Abstract

**CONTEXT AND OBJECTIVE:**

Breast cancer accounts for approximately one quarter of all cancers in females. Estrogen and progesterone receptor testing has become an essential part of the clinical evaluation of breast carcinoma patients, and accurate results are critical in identifying patients who may benefit from hormone therapy. The present study had the aim of investigating the concordance of the results from hormone receptor tests between a reference laboratory and local (or community) laboratories in Brazil.

**DESIGN AND SETTING:**

Retrospective study at a reference pathology laboratory.

**METHODS:**

The concordance in the results from hormone receptor tests between a reference laboratory and 146 local laboratories in Brazil was compared in relation to 500 invasive breast carcinoma cases, using immunohistochemistry.

**RESULTS:**

There was concordance in 89.4% (447/500 cases) and 85.0% (425/500 cases) of the results from estrogen (k = 0.744, P < 0.001) and progesterone (k = 0.688, P < 0.001) receptor tests, respectively, between local and reference laboratories. This was similar to findings in other countries. The false negative rates from estrogen and progesterone receptor tests in local laboratories were 8.7% and 14.4%, respectively. The false positive rates from estrogen and progesterone receptor tests in local laboratories were 15.5% and 16.0%, respectively.

**CONCLUSION:**

Technical and result interpretation issues may explain most of the discordances in hormone receptor testing in local laboratories. Validation of estrogen and progesterone receptor tests at local laboratories, with rigorous quality control measures, is strongly recommended in order to avoid erroneous treatment of breast cancer patients.

## RESUMO

**CONTEXTO E OBJETIVO:**

O carcinoma de mama é responsável por cerca de um quarto de todos os cânceres em mulheres. O teste de receptores de estrógeno e progesterona se tornou parte essencial da avaliação clínica de pacientes com carcinoma de mama; assim, resultados precisos são fundamentais para identificação de pacientes que podem se beneficiar da terapia hormonal. O presente estudo tem por objetivo investigar a concordância nos resultados do teste de receptores hormonais entre um laboratório referência e laboratórios locais (ou comunitários) do Brasil.

**TIPO DE ESTUDO E LOCAL:**

Estudo retrospectivo em laboratório referência em patologia no Brasil.

**MÉTODOS:**

A concordância nos resultados dos testes de receptores hormonais entre um laboratório referência e 146 diferentes laboratórios locais brasileiros foi comparada em 500 casos de carcinoma invasivo de mama através da imunoistoquímica.

**RESULTADOS:**

Houve concordância de 89,4% (447/500 casos) e 85,0% (425/500 casos) nos resultados dos testes de receptores de estrógeno (k = 0,744, P < 0,001) e progesterona (k = 0,688, P < 0,001), respectivamente, entre laboratórios locais e referência, similar à descrita em outros países. A taxa de resultados falso-negativos nos testes de receptores de estrógeno e progesterona em laboratórios locais foi de 8,7% e 14,4%, respectivamente. A taxa de resultados falso-positivos nos testes de receptores de estrógeno e progesterona em laboratórios locais foi de 15,5% e 16,0%, respectivamente.

**CONCLUSÃO:**

Questões técnicas e de interpretação dos resultados podem explicar a maior parte das discordâncias nos testes de receptores hormonais em laboratórios locais. A validação dos testes de receptores de estrógeno e progesterona pelos laboratórios locais com medidas de controle de qualidade rigorosas é fortemente recomendada de modo a evitar o tratamento errôneo de pacientes com carcinoma de mama.

## INTRODUCTION

Breast cancer is one of the most common human neoplasms, accounting for one quarter of all cancers in females.^1^ Hormone therapy is frequently used in breast carcinoma treatment because it reduces the relative risk of recurrence by more than 50% in patients with hormone-sensitive tumors, thus leading to significant improvements in survival. For these reasons, determination of estrogen receptor (ER) and progesterone receptor (PgR) status has become an essential part of the clinical evaluation of all breast carcinoma patients, and accurate results are critical in identifying patients who may benefit from hormone therapy.^2–8^ Immunohistochemistry (IHC) is currently the most common method used for determining ER and PgR status. Low cost and applicability to routinely processed and archived tissue samples are the main advantages of IHC. However, discordances in ER and PgR testing have been reported in the literature, and they have been mostly correlated with technical issues, including fixative and fixation issues, immunohistochemical methodology and diversity of interpretation of results.^9–29^

## OBJECTIVE

Considering that the accuracy of ER and PgR testing in breast carcinoma is extremely important in selecting the hormone therapy, the present study had the aim of investigating the concordance of the results from ER and PgR tests using IHC between a reference laboratory (Pathology Consultancy, Botucatu, São Paulo, Brazil) and local (or community) laboratories in Brazil.

## METHODS

### Institutional certifications

This study was approved by the Scientific Committee of the Department of Pathology, Faculdade de Medicina da Universidade de São Paulo (FMUSP), and by the Ethics Committee for Research Projects of Hospital das Clínicas (HC), FMUSP (CAPPesq, protocol n^o^ 1238/09).

### Validation at the reference laboratory

For any clinical assay to be validated, the results need to be compared with a standard. For ER and PgR testing, the recommended approach towards validation is based on comparison of the assay results with results obtained by another laboratory using a testing method that has been previously validated against the clinical outcome or using proficiency-testing material that has been validated by showing a consensus of results among multiple laboratories in a peer group (which must include laboratories with validated assays). ER and PgR IHC assays that are not subjected to direct clinical validation may be validated by showing at least 90% agreement for positive results and at least 95% agreement for negative results (a minimum of 20 positive and 20 negative specimens are required) with testing performed on the same material in another laboratory that provides written attestation that it is in conformity with the testing requirements of the American Society of Clinical Oncology (ASCO) and College of American Pathologists (CAP) and that offers fully validated ER and PgR assays.^6,7,30^

Pathology Consultancy, a reference laboratory located in Botucatu, São Paulo State, Brazil, performs approximately 7,000 ER and PgR assays by means of IHC annually. In order to further validate the ER and PgR testing by Pathology Consultancy, we compared the results from ER and PgR IHC assays between a CAP-accredited laboratory that performs fully validated ER and PgR testing (PhenoPath Laboratories, Seattle, Washington, United States) and Pathology Consultancy. For validation purposes, 255 invasive breast carcinoma samples (a 2-mm tissue core for each sample) were distributed over nine tissue microarray (TMA) blocks. Unstained 3-μm-thick histological sections from each TMA block were obtained and used to determine the ER and PgR status using IHC, both in PhenoPath Laboratories and in Pathology Consultancy.

In Pathology Consultancy, the sections were deparaffinized in xylene and rehydrated in graded alcohols and phosphate-buffered saline (PBS). The sections were then subjected to antigen retrieval in a pressure pan using citric acid (0.21%, pH 6.0) for a total of eight minutes, followed by a 20-minute cool-down period at room temperature. Subsequently, the slides were incubated overnight with the primary antibody. The SP1 rabbit monoclonal antibody (code RM-9101-S, Thermo Fisher Scientific, Fremont, California, United States; dilution 1:1000) was used for ER testing. The PgR 636 mouse monoclonal antibody (code M3569, Dako, Carpinteria, California, United States; dilution 1:1600) was used for PgR testing. After incubation with the primary antibody, the slides were washed with PBS, and incubated with Novolink polymer (code 7161, Novocastra Laboratories, Newcastle upon Tyne, United Kingdom) for 30 minutes. Diaminobenzidine was used as the chromogen, and the sections were then counterstained with hematoxylin and coverslipped. The same antibodies (SP1 for ER and PgR 636 for PgR) were also used at PhenoPath Laboratories, using fully validated technical procedures.

The results from the ER and PgR assays were compared between PhenoPath Laboratories and Pathology Consultancy, in line with the most recent consensus of ASCO and CAP: the ER and PgR tests were considered positive if at least 1% of the tumor nuclei in the samples tested were positive, irrespective of the intensity of staining.^6,7,30^

### Case selection for comparison between reference and local laboratories

The study group included 500 consecutive cases of invasive breast carcinoma received in 2008 and 2009 for ER and PgR testing by Pathology Consultancy that had previously been tested in 146 different local laboratories in all geographic regions of Brazil. These local laboratories consisted of community-based laboratories with low volumes of ER and PgR testing. The cases included in the study were sent to the reference laboratory by oncologists for confirmation of breast cancer diagnosis and for ER and PgR retesting.

All the cases were morphologically reviewed in the reference laboratory by at least two different pathologists in order to confirm the diagnosis of invasive breast carcinoma and to classify the tumors in accordance with the World Health Organization (WHO) classification.^1^

Information regarding the ER and PgR testing performed by the local laboratories was retrieved from the immunohistochemical reports, including the geographic location of the laboratory, details about fixation of the specimen (fixative used and fixation time), type of breast carcinoma tested (in situ or invasive carcinoma), use of tissue controls, IHC methodology (antibodies, antigen retrieval technique and detection system specifications), ER and PgR results and interpretation criteria for reporting the results. Paraffin-embedded breast carcinoma tissue from all cases was used for ER and PgR testing in the reference laboratory. The same paraffin blocks from the local laboratories were used. IHC was used to determine ER and PgR status, as previously described. In accordance with the most recent consensus of ASCO and CAP,^6,7^ the ER and PgR results were considered positive if at least 1% of the tumor nuclei in the samples tested were positive, irrespective of the intensity of staining. The ER and PgR results were interpreted blindly by experienced pathologists, without knowledge of the previous results defined by local laboratories.

### Statistical analysis

The reference and local laboratories results for ER and PgR were compared using Pearson's chi-square (χ^2^) test. Kappa statistics were used as a concordance measurement. Sensitivity, specificity, false negative rate, false positive rate, positive predictive value, negative predictive value, overall accuracy and the Youden index were calculated. The significance level used in all tests was 5%.^31–34^

## RESULTS

### Validation by the reference laboratory

As previously presented, 255 invasive breast carcinomas samples were tested for ER and PgR for validation purposes. In the CAP-accredited laboratory (PhenoPath Laboratories), 172 samples were considered positive and 83 were negative for ER; 164 samples were considered positive and 91 were negative for PgR. The overall concordance between PhenoPath Laboratories and Pathology Consultancy was 98.8% (252/255 samples) and 98.4% (251/255 samples) for ER and PgR, respectively. For positive results, the concordance was 100.0% for both ER and PgR. For negative results, the concordance was 96.4% for ER and 95.6% for PgR. These results fully validate the ER and PgR testing performed at Pathology Consultancy, according to recent recommendations for validating ER and PgR IHC assays.^6,7,30^

### Comparison of results between reference and local laboratories

The 500 cases of invasive breast carcinoma included in the study were classified as invasive ductal carcinoma that was not otherwise specified (93.8%) and invasive lobular carcinoma (2.6%). Micropapillary, apocrine, tubular, papillary, metaplastic and mucinous carcinomas were rarely found (3.6%), as shown in Table 1. Cases from all geographic regions of Brazil were represented. Most of the cases were from the southeastern region (70.0%), followed by the southern (12.8%), northeastern (8.0%), central-western (6.6%) and northern (2.6%) regions. The total number of cases from each local laboratory ranged from 1 to 41 cases.

**Table 1.** Histological classification of invasive breast carcinomas according to World Health Organization (WHO), 2003

Histological type	No. cases	%
Ductal carcinoma, NOS	469	93.8
Lobular carcinoma	13	2.6
Micropapillary carcinoma	5	1.0
Apocrine carcinoma	4	0.8
Tubular carcinoma	4	0.8
Papillary carcinoma	3	0.6
Metaplastic carcinoma	1	0.2
Mucinous carcinoma	1	0.2
Total	500	100.0

NOS = not otherwise specified.

Information about specimen fixation (including the fixative used and fixation time) was found in only 32 reports (6.4%) from local laboratories, and all of them reported that formalin was the fixative. Fixation time was not found in any of the reports. Most reports (67.6%) did not provide any information about the type of breast carcinoma (*in situ* or invasive carcinoma) that was tested in IHC assays. Only 209 reports (41.8%) provided information on the use of tissue controls for IHC assays.

The antibody used for ER testing by the local laboratories was not specified in 169 cases (33.8%). When the information regarding ER antibody specification was available, 1D5 (53.8%), 6F11 (24.2%) and SP1 (13.3%) antibodies were used most frequently; CC4-5 was employed in 16 (4.8%) cases, followed by SP4 (2.1%), RBT11 (1.2%) and LH2 (0.6%). The antibody used for PgR testing by the local laboratories was not specified in 194 cases (38.8%). When the information regarding PgR antibody specification was available, PgR 636 (36.3%), 1A6 (29.1%), 16 (13.7%) and 312 (10.4%) antibodies were used most frequently; NCL was used in 7 cases (2.3%), followed by PR-2C5 (1.6%), SP2 (1.6%), 1A7 (1.0%), RBT22 (1.0%), sp21 (1.0%), Y85 (1.0%), M3569 (0.7%) and PGR-NCL (0.3%).

The antigen retrieval method was not specified in 198 reports (39.6%). When available, heat-induced antigen retrieval was used. The detection system was not specified in 128 reports (25.6%). When available, LSAB (labeled streptavidin biotin) was used most frequently (60.5%), followed by ABC (avidin biotin complex) (15.3%), “polymer” (15.3%), EnVision™ (5.9%), ADVANCE™ HRP (2.4%) and PAP (peroxidase anti-peroxidase) (0.6%).The comparison of ER and PgR results between the reference and local laboratories is shown in Tables 2 and 3.

**Table 2.** Distribution of estrogen receptor (ER) results between reference and local laboratories (kappa statistic k = 0.744, P < 0.001; Pearson's chi-square association test χ^2^ = 273.889, P < 0.0001)

			Reference laboratory ER result		
			Negative	Positive	Total
Local laboratory ER result	Negative	n	120	31	151
		%	84.5	8.7	30.2
	Positive	n	22	327	349
		%	15.5	91.3	69.8
	Total	n	142	358	500
		%	28.4	71.6	100.0

**Table 3.** Distribution of progesterone receptor (PgR) results between reference and local laboratories (kappa statistic k = 0.688, P < 0.001; Pearson's chi-square association test χ^2^ = 234.490, P < 0.0001)

			Reference laboratory PgR result		
			Negative	Positive	Total
Local laboratory PgR result	Negative	n	163	44	207
		%	84.0	14.4	41.4
	Positive	n	31	262	293
		%	16.0	85.6	58.6
	Total	n	194	306	500
		%	38.8	61.2	100.0

Using kappa statistics as a concordance measurement (κ = 0.744; P < 0.001; 95% confidence interval 0.679-0.809), the overall accuracy of ER testing in local laboratories was 89.4% (447/500 cases). ER-positive and negative results were concordant between the reference and local laboratories in 91.3% (327/358) and 84.5% of the cases (120/142), respectively. Pearson's chi-square (χ^2^) association test for ER results between the reference and local laboratories revealed χ^2^ = 273.889 (P < 0.0001), which indicates a significant association in the results. The sensitivity, specificity, false negative rate, false positive rate, positive predictive value, negative predictive value and Youden index of ER testing in local laboratories were 91.3%, 84.5%, 8.7%, 15.5%, 93.7%, 79.5% and 75.8%, respectively. Using kappa statistics as a concordance measurement (κ = 0.688; P < 0.001; 95% confidence interval 0.623-0.753), the overall accuracy of PgR testing in local laboratories was 85.0% (425/500 cases). PgR-positive and negative results were concordant between the reference and local laboratories in 85.6% (262/306) and 84.0% of the cases (163/194), respectively. Pearson's chi-square (χ^2^) association test for PgR results between the reference and local laboratories revealed χ^2^ = 234.490 (P < 0.0001), which indicates a significant association in the results. The sensitivity, specificity, false negative rate, false positive rate, positive predictive value, negative predictive value and Youden index of PgR testing in local laboratories were 85.6%, 84.0%, 14.4%, 16.0%, 89.4%, 78.7% and 69.6%, respectively.

Unfortunately, there was no comment concerning the criteria used for interpretation of the ER and PgR results in 155 reports (31.0%) and 162 reports (32.4%) from the local laboratories, respectively. When such information was available, the reports from local laboratories stated that the interpretation of the ER results was based on quantification of cells only (59.7%), both quantification of cells and intensity of immunostaining (39.7%), or intensity of immunostaining only (0.6%). The interpretation of PgR results was based on cell quantification only (60.0%), both cell quantification and the intensity of immunostaining (39.7%), or the intensity of immunostaining only (0.3%). No specification regarding the minimal percentage of cells necessary for the test to be considered positive was found in any report from the local laboratories.

## DISCUSSION

Brazil has a population of approximately 190 million people.^35^ The incidence of breast cancer in Brazil is about 50,000 new cases per year,^36^ and it is considered to be an important public health problem.

Hormone receptor status should be defined in all newly diagnosed, invasive breast carcinomas as well as in recurrences, in order to determine patient eligibility for hormone therapy, which provides substantial survival benefit for patients with hormone-positive tumors. Accurate determination of ER and PgR status is, therefore, critical for ensuring that patients receive appropriate therapy.^2–8,30^ However, discordances in hormone receptor testing using IHC have been reported in different laboratories from several countries, and these probably relate to technical issues, including delayed or inadequate fixation, non-optimized antigen retrieval and diversity of interpretation and reporting of results.^9–29^

In 2000, Rhodes et al.^10^ demonstrated that there was considerable variability between laboratories (200 laboratories in 26 countries) regarding ER results, especially in relation to detection of breast cancers with low ER positivity, with a false-negative rate ranging from 30% to 60%. This variability between laboratories probably related to differences in IHC methodology, according to these authors.

In 2001, in a study that involved 105 laboratories, the same authors showed that the efficiency of the antigen retrieval step was the single most important contributory factor influencing the overall reproducibility of the hormone assays. Reliable assays were found in the majority of centers known to have clinically validated results. Inadequate assay sensitivity, with subsequent weak staining, was the main cause of poor and variable results at laboratories that used microwave antigen retrieval; heating times that were too short were identified as the principal contributory factor. Extension of the heating time resulted in significant improvement regardless of all other variables in the immunohistochemical protocol. They also stated that continual participation in external quality assessment programs was an effective way to identify and improve variables that influence the reliability of immunohistochemical assays for ER and PgR determination, thereby assisting in technical validation and standardization.^14^

Regitnig et al.^15^ investigated the variability in the results from ER and PgR testing performed by different laboratories in Austria. They found that the variability in the results was greater when participants used their own IHC staining method. In 2007, Viale et al.^22^ evaluated locally versus centrally assessed ER and PgR status among a significant number of breast cancer patients. Out of 105 tumors that were considered to be ER-negative in local laboratories, 81 were found to have positive cell rates of at least 1% at the central laboratories. Out of 6,100 tumors that were found locally to be ER-positive, 66 were found to have no staining centrally. The discordance was more marked for PgR than for ER. Because of these results, Regitnig et al.^15^ recommended that the ER and PgR status should be reviewed in central laboratories whenever possible. Badve et al.^23^ showed a concordance rate of 90% in ER testing between local and central laboratories. For PgR, the concordance between local and central laboratories was 84%. A recent inquiry into ER testing practices in Canada revealed that approximately one third of 1,023 ER assays were scored falsely negative when retested in a central laboratory. The possible causes of such discrepancies related to turnover and lack of relevant training of pathologists and technologists, lack of appropriate quality assurance methods, and inadequate quality control policies and practices. The false negative ER assays were found to have poor fixation, negative internal controls, and/or absent internal controls.^26^ In 2009, Gelber et al.^27^ reported that 4.3% of the tumors that tested ER-positive in local laboratories were found to be negative (false positive) on central review. More than 20% of the tumors that tested locally as ER-negative were shown to exhibit at least some expression of ER (false negative) on central reference laboratory review. Table 4 presents a comparison of the concordance results for ER and PgR between laboratories reported in the literature and by the present study.

**Table 4.** Comparison of the concordance results for estrogen (ER) and progesterone (PgR) receptors between reference/central and local laboratories reported in the literature and by the present study

Authors, country and year of publication	ER concordance	PgR concordance
Viale et al.,^22^ 25 countries, 2007	6058/6205 (97.6%)	4202/5237 (80.2%)
Badve et al.,^23^ USA, 2008	694/769 (90.2%)	649/769 (84.4%)
Gelber et al.,^27^ several countries, 2009	4323/4931 (87.6%)	-
Wludarski et al., Brazil, 2011 (present study)	447/500 (89.4%)	425/500 (85.0%)

Pathology Consultancy is considered to be a reference laboratory in Brazil because of its high volume of ER and PgR testing (approximately 7,000 assays every year). In the present study, we compared the results from ER and PgR testing on 500 invasive breast carcinomas between a reference laboratory and 146 different local laboratories in Brazil. Local, low-volume laboratories from all geographic regions of Brazil were represented. Even though the overall concordance of the results from ER and PgR assays between the reference and local laboratories was high (89.4% for ER and 85.0% for PgR) and the ER-positive assays were concordant in 91.3% of the cases, the ER-negative assays were concordant in only 84.5%, a result that is lower than the minimum recommended. The same was true for the PgR-positive and negative results. They were concordant in 85.6% and 84.0% of the cases, respectively. As previously stated, at least 90% and 95% agreement for positive and negative results is recommended.^6,7,30^ The false negative rates for ER and PgR testing in the local laboratories were 8.7% and 14.4%, respectively. This means that patients who are misclassified as having ER-negative tumors are denied the potential benefit of hormone treatment. The false positive rates for ER and PgR testing in the local laboratories were 15.5% and 16.0%, respectively. This means that patients who were misclassified as having ER-positive tumors will be exposed unnecessarily to the risks and costs of ineffectual treatment. The risks include a decrease in bone density with an increased fracture risk, an increased risk of thromboembolic events and an increased risk of endometrial cancer.^37^ Fixation issues, lack of use of tissue controls, diverse immunohistochemical protocols (i.e. different antibodies and antigen retrieval methods) that were probably not validated as recommended by ASCO and CAP, and heterogeneity in interpretation criteria for reporting the results could explain most of the ER and PgR testing discordances between the local and reference laboratories in Brazil.

## CONCLUSION

This study presents relatively high concordance of the results from ER and PgR testing between local laboratories and a reference laboratory in Brazil, similar to findings from other countries. However, false-negative and false-positive results occur, which may relate to technical and interpretation issues. These results may be associated with erroneous treatment of breast cancer patients. We believe that validation of ER and PgR testing and standardization of the interpretation of the results, with rigorous quality control measures at local laboratories, are crucial. Moreover, reference laboratories could assist in validating local laboratories’ ER and PgR assays.
